# How Does Adolescents’ Openness to Diversity Change Over Time? The Role of Majority-Minority Friendship, Friends’ Views, and Classroom Social Context

**DOI:** 10.1007/s10964-020-01329-4

**Published:** 2020-10-31

**Authors:** Sevgi Bayram Özdemir, Metin Özdemir, Katja Boersma

**Affiliations:** 1 Center for Lifespan Developmental Research (LEADER), School of Law, Psychology and Social Work, Örebro University, 701 82 Örebro, Sweden; 2 Center for Health and Medical Psychology (CHAMP), School of Law, Psychology and Social Work, Örebro University, 701 82 Örebro, Sweden

**Keywords:** Openness to diversity, Cross-ethnic friendship, Peers, Classroom ethnic composition, School climate

## Abstract

Young people are growing up in increasingly “super-diverse” societies, and show variations in how they approach diversity and embrace differences. Developing a good understanding of why some youth appreciate and value diversity whereas others do not is crucial in identifying ways to promote social interactions among different groups in broader society. The current study examined whether adolescents follow different trajectories in their views on diversity, and identified possible factors behind how they change over time. The sample included 1362 adolescents residing in Sweden (*M*_*age*_ = 13.18, *SD* = 0.43, 48% girls). Adolescents reported on their openness to diversity and classroom social climate. The peer nominations method was used to measure majority-minority friendship, and friends’ views on diversity. Latent growth analysis showed that adolescents, on average, became more open to diversity over time, but with clear heterogeneity. Three distinct trajectories were identified as: *high-increasing*, *average-increasing*, and *average-declining*. Relative to the high-increasing group, the other two were more likely to be male and immigrant. Relative to the high-increasing group, adolescents on the average-increasing trajectory perceived their classroom climate as less cooperative, while the adolescents on the average-declining trajectory were less likely to have friends with positive views on diversity. The findings suggest that schools may serve as a shared ground for promoting openness to diversity.

## Introduction

Due to significant waves of immigration and globalization, today’s youth are growing up in increasingly “super-diverse” societies. Some youth appreciate this diversity and take the opportunity to engage with others and embrace differences. Some others, by contrast, are more hesitant or even sometimes resistant in interacting with people who are different from themselves. This social reality highlights the importance of understanding the factors that might facilitate youth’s ability to embrace differences, to appreciate and value diversity, and eventually to interact with diverse peers in positive and effectual ways. Developing such an understanding is particularly critical in adolescence because the bases of global competences are formed during this developmental period, and are likely to influence young people’s adulthood views and their social interactions with diverse groups in the broader society. In light of these issues and current gaps in knowledge, the present study examined: (1) how adolescents’ views on diversity change during adolescence, (2) whether adolescents follow different trajectories in their views, and (3) the extent to which adolescents’ peer contexts and experiences in school contribute to the pathway they have taken. These research questions were investigated within the Swedish cultural context.

Diversity is increasing rapidly in Sweden; new ethnic and cultural groups are entering the country for various reasons, such as escaping from war and political oppressions, getting a better education, and finding a better job. In the last two decades alone, the proportion of Swedish residents born in a country other than Sweden has increased from 11% to 19%. Today, just over 2.4 million of Sweden’s entire population (of about 10 million) were born in a country other than Sweden or have two foreign-born parents (https://www.scb.se). Sweden is by no means unique, but similar population transformations are also noticeable in other European countries. Sweden has been presented as one of the most successful European countries in terms of integration (MIPEX 2015) and of citizens’ views on immigrants in cross-national surveys (European Social Survey 2014). But, despite its promising record on diversity and migration policies, Swedish society has also experienced increased polarization due to the immigration crisis and growing anti-immigrant ideologies in the European Union. Such polarization might also have consequences for how youth approach diversity and embrace differences, and highlights the need to integrate developmental and contextual perspectives to uncover how young people’s views on diversity change over time.

### What are the Indicators of Openness to Diversity?

Openness to diversity has been conceptualized as “an awareness and potential acceptance of both similarities and differences in others” (Fuertes et al. 2000, p. 158), and can be expressed through one’s beliefs, feelings, and behaviors. Starting from early ages, individuals living in “super-diverse” societies are surrounded by others who are different from themselves regarding how they look, what they believe in, and how they live their social lives (Titzmann and Jugert 2019). This diversity brings greater opportunity to engage with an array of perspectives. However, diversity itself is not enough for an individual to benefit from growing up in a diverse environment. Individuals vary from each other in the extent to which they are interested in, and capable of interacting with others who are different from themselves. Individual differences among people and variations in their socialization contexts may explain why they follow different pathways. In line with these arguments, previous research has identified several individual (Gerson and Neilson 2014; Han 2017) and contextual (Flowers and Pascarella 1999; Pascarella et al. 1996) factors that are associated with openness to diversity. For example, young adults who have the ability to empathize with others or who adopt a sociocentric perspective in their moral reasoning (i.e., focus on fairness, social justice, and equality) have been found to be more open to diversity. By contrast, those who experience identity confusion or who hold an idiocentric perspective in their reasoning (i.e., who are self-oriented, or value competition) tend to perceive others who are different from themselves as a threat (Gerson and Neilson 2014). Further, social influence has also been shown to be involved in the formation of openness to diversity. Specifically, being in a non-discriminatory college environment (Flowers and Pascarella 1999; Pascarella et al. 1996), interacting with a diverse group of peers (Antonio 2001), or having faculty members who advocate respect on the part of students for diverse viewpoints (Ryder et al. 2016) is associated with greater willingness to embrace differences among university students. Together, these findings indicate that individual differences (e.g., empathy and identity formation) and socialization context (e.g., being in an inclusive or non-discriminatory context) may determine how individuals approach others’ perspectives and embrace differences.

Despite an increasing interest in understanding the factors that might play a role in why some people are open to diversity whereas others are not, there are two main limitations of the current literature. *First*, the available studies mostly focus on young adults, namely university students; not much is known about how young people approach diverse perspectives during their adolescence, except from studies examining adolescents’ intergroup relationships (Rivas‐Drake et al. 2019) and attitudes toward immigrants (van Zalk and Kerr 2014; Zingora et al. 2020). Critical cognitive and social changes occur during adolescence. Specifically, young people explore their self and identity (McLean and Syed 2015), and form their views about out-group members. In addition, they become more cognitively mature, which allows them to have advanced perspective-taking skills and prosocial moral reasoning in their social interactions (Eisenberg et al. 2005). Further, peers become an increasingly more important part of adolescents’ social worlds during this developmental period. Relatedly, adolescents demonstrate a greater sensitivity to peer relationships and are more open to peer influence (Brown and Larson 2009). Thus, understanding how adolescents’ views on diversity are shaped during adolescence and what promotes or hampers the development of openness to diversity, should be informative in identifying ways to intervene with risk factors early on, and in promoting interactions among diverse groups in the broader society.

*Second*, the heterogeneity among youth regarding how they change in their views has not been systematically examined, even in studies that examine the change over time in attitudes toward immigrants during adolescence (van Zalk and Kerr 2014; Wölfer et al. 2016). Youth on average may become more tolerant toward outgroup members (van Zalk and Kerr 2014), or more open to diverse perspectives in parallel with increasing cognitive maturation and use of advanced perspective-taking skills (Eisenberg et al. 2005), as they get older. However, not all youth change in the same direction. Considering inter-individual differences may help in the identification of adolescents who follow different trajectories in their views on diversity over time and detect factors that might play a role in why they follow different pathways. To address these issues, the present study examined whether young people follow different trajectories in their views on diversity during adolescence (from age 13 to 15), and investigated whether adolescents’ peer (e.g., majority-minority friendship and friends’ openness to diversity) and school contexts (e.g., classroom ethnic composition and perception of positive classroom social climate) increase their openness to diversity over time.

### The Role of Majority-Minority Friendship

The contact hypothesis stipulates that the availability of social contact between people from different backgrounds is the base for the development of intergroup relationships. Specifically, Allport (1954) argues that contact between groups is effective in promoting tolerance and reducing prejudice when people have equal status, share common goals, and cooperate with each other. Following the premises of the contact hypothesis, a growing body of research has examined whether cross-ethnic friendship (which is assumed to have the characteristics of social contact as highlighted by Allport) has an impact on adolescents’ intergroup attitudes (see Davies et al. 2011; Pettigrew and Tropp 2006 for recent meta-analytic reviews). Across cross-sectional and longitudinal studies, it has been shown that cross-ethnic friendships promote adolescents’ positive feelings and attitudes toward outgroup members (Chen and Graham 2015; Kelleghan et al. 2019), and also their willingness to engage in different communal activities, such as attending school parties together, eating lunch together, or visiting each other’s homes (Chen and Graham 2015). Such relationships also reduce intergroup anxiety, and, in turn, counteract the development of prejudiced feelings (Binder et al. 2009). Together, the available studies consistently suggest that cross-ethnic friendships are key to having and maintaining positive intergroup attitudes.

Despite an increase in studies examining the potential role of cross-ethnic contact in the development of intergroup attitudes, only limited knowledge is available regarding whether forming such relationships also promotes young people’s ability to embrace differences and to appreciate and value diversity without referencing any specific group. Two competing conceptual arguments may be plausible here. One argument is that interacting with diverse peers can result in having “conversations about controversial or value laden issues that may engender a change in perspective or opinion” (Pascarella et al. 1996, p. 188). Relatedly, young people with cross-ethnic friends might have more opportunity to engage in diverse perspectives, social relations, and life styles. Such opportunities may help them become cognitively and behaviorally flexible in their approach to diverse views and tolerant of them. In line with these arguments, studies focusing on college students have shown that the more students interact with diverse peers (i.e., peers of different racial, cultural, religious, national origin, and economic backgrounds), the more they become open to diversity (Pascarella et al. 1996; Whitt et al. 2001). The alternative argument is that inter-ethnic contact might be contextually bounded in its effect on young people’s views and attitudes. That is, a young person who has a friend of migrant background might develop positive attitudes toward immigrants (Feddes et al. 2009). However, such cross-ethnic interaction might not have any spillover effect and not necessarily promote that person’s openness to diversity in general. To test these two competing arguments, the present study examined the extent to which having an immigrant friend (or a Swedish friend in the case of immigrants) increases openness to diversity among adolescents over time.

### The Role of Friends’ Views on Diversity

Adolescents spend a great amount of their time with their peers in and outside of school. Peers also become an important frame of reference and provide useful social information during this developmental period, and thereby have a broad influence on how adolescents view and engage with others. Different, but interrelated, theoretical arguments (e.g., from shared reality theory and group norm theory) have been used to explain why adolescents are influenced by their peers in forming their views and behaviors (Hardin and Conley 2001; Sherif and Sherif 1953). According to these theories, people tend to develop a common understanding with others on different matters on the ground that the formation of shared understanding may help them establish and maintain a social relationship. In addition, they often experience pressure to conform to larger social and group norms, and try to avoid contradicting group norms to avoid social sanctions. Such desires and tendencies might be some of the underlying reasons why adolescents are likely to internalize the views and attitudes prevalent in their peer group.

Supporting these arguments, research has shown that peers may serve as socializing agents in the formation and continuation of intergroup relations (Tropp et al. 2016). That is, the peers whom adolescents interact with—and these people’s views on out-group members—may influence what adolescents think about out-group members (Rivas‐Drake et al. 2019; van Zalk et al. 2013; Zingora et al. 2020) and how they interact with them (Titzmann et al. 2015; Tropp et al. 2016). For example, recent studies have shown that adolescents become more tolerant over time (van Zalk and Kerr 2014), or develop a positive orientation toward intergroup relations (Rivas‐Drake et al. 2019; Zingora et al. 2020), when they themselves have friends with positive views on intergroup relations. Importantly, this socialization effect holds even after ruling out the possible selection effect. In sum, these studies suggest that youth tend to share similar attitudes to their peers, and tend to act in line with social norms in their peer settings.

Despite an increasing interest in the role of peers in adolescents’ views and attitudes, the studies available have primarily examined whether peers matter in the formation of youth’s group-specific attitudes (e.g., toward immigrants). To our knowledge, no previous study has examined the extent to which peers play a role in youth’s views on diversity in general. Having such an understanding would help us establish whether peers are also influential in the formation of non-group-specific attitudes.

### The Role of Classroom Ethnic Composition

School is an important social arena for youth to meet with peers of different backgrounds. However, not all youth attend schools of similar ethnic or cultural composition. The socio-ethnic compositions of schools vary to a larger extent in parallel with characteristics of their neighborhoods. Thus, adolescents’ opportunity for being exposed to peers of diverse background, and relatedly their views on diversity, might also differ according to the ethnic composition of their school. Two main conceptual arguments have been used in the literature to explain the ways in which ethnic diversity in school has an impact on intergroup attitudes. Following contact theory (Allport 1954), the first conceptual perspective posits ethnic diversity as an avenue for improving intergroup relationship. By contrast, following group competition theory (Coenders et al. 2004), the second perspective suggests that diversity can threaten the power of the majority, and thus may result in social tension. The available empirical studies have findings that support both perspectives. For example, adolescents in ethnically heterogenous classrooms were found to support multiculturalism (van Geel and Vedder 2011), and to hold tolerant attitudes toward immigrants (Janmaat 2014), or to outgroup members (Burgess and Platt 2020). By contrast, several studies provided null (Bekhuis et al. 2013) or contrasting (Vervoort et al. 2011) effects of classroom ethnic composition. For example, in a large-scale study focusing on adolescents in the Netherlands, the relations between classroom ethnic composition (defined as the proportion of non-western ethnic minorities in class) and adolescents’ inter-group attitudes (operationalized as the characteristics that students consider people of the in-group and out-group to have in general) was investigated. It was found that students in classrooms with a large share of ethnic minority students (more than 50%) had more negative out-group attitudes than those in classrooms with no or a small proportion of ethnic minority students (Vervoort et al. 2011). In sum, these findings suggest that the effect of classroom ethnic composition on adolescents’ intergroup attitudes is not clear cut. A similar conclusion might apply to the possible role played by classroom ethnic composition in youth’s openness to diversity. This issue was examined in the current study by testing the extent to which being in an ethnically diverse classroom favors or hinders young people’s openness to diversity during adolescence.

### The Role of Perceived Classroom Social Climate

Classrooms may differ from each other regarding their common values and norms, how students interact with each other, and how teachers approach and treat the students. The social dynamics in classrooms have implications for students’ psychosocial functioning (Wang 2009) and academic growth (Reyes et al. 2012). Recent studies have also shown that common norms and values in a classroom setting may have an impact on students’ inter-group attitudes (Gniewosz and Noack 2008; Isac et al. 2012; Schachner et al. in press) and relationships (Bayram Özdemir and Özdemir 2020; Bayram Özdemir et al. 2018; Schachner et al. 2015). For example, a study focusing on adolescents in Germany investigated the influence of different indicators of the classroom climate on adolescents’ intolerance in their attitudes toward foreigners. It was found that students’ individual perception of fairness in school was related to having more tolerant attitudes, while their perception of achievement pressure in school was related to having more intolerant attitudes (Gniewosz and Noack 2008). Similarly, a recent cross-national study showed that when adolescents perceived their classroom as open for discussions, they held more tolerant attitudes (Isac et al. 2012). Together, these findings suggest that the perception of fairness in a classroom setting or having opportunities for open discussions may promote feelings of justice among adolescents and the recognition of different perspectives. On the other hand, high levels of achievement pressure may lead to competition among students, and thereby direct students’ focus toward differences rather than similarities, which may lead to non-optimal conditions for cross-ethnic contact.

Not only students’ views on the norms and values in a classroom setting, but also their perceptions about social interactions within the classroom may play a role in how they perceive students who are different from themselves. Specifically, if students perceive their classroom contexts as socially cohesive and cooperative (e.g., everyone helps each other, cooperates well, and no one feels left out), the environment may facilitate the development of “we-ness” and contribute to a common in-group identity (Gaertner et al. 1993). Students may then not perceive difference as a threat toward themselves, but rather as an opportunity to learn more about diverse perspectives. In line with these arguments, it was expected that being in a socially cohesive and cooperative classroom environment would contribute to the development as well as maintenance of greater openness to diversity among the adolescents.

## The Current Study

Using three-year longitudinal data, the current study aimed to address two important questions. The first was to explore whether adolescents follow different developmental trajectories regarding their views on diversity from age 13 to 15. Based on previous research (Eisenberg et al. 2005; van Zalk and Kerr 2014), it was expected that adolescents on average might display greater openness to diversity as they get older, due to cognitive maturation and development in perspective-taking skills and prosocial moral reasoning. However, it was also expected that not all adolescents change in their views on diversity in the same direction, since there are inter-individual differences among adolescents. Thus, it was hypothesized that multiple distinct trajectories would emerge. The second was to examine whether adolescents’ peer (i.e., majority-minority friendship and friends’ openness to diversity) and school contexts (i.e., classroom ethnic composition and perception of classroom social climate) play a role in how they change over time. Based on theoretical reasoning (Hardin and Conley 2001; Sherif and Sherif 1953) and previous empirical research (Gniewosz and Noack 2008; Rivas‐Drake et al. 2019), it was expected that adolescents who socialize with open minded friends or who perceive their classroom environment as cooperative and socially cohesive would be more likely to be open to diverse perspectives and to embrace differences to a greater extent over time. Regarding the roles of majority-minority friendship and classroom ethnic composition in youth’s views on diversity, there are competing conceptual arguments and empirical findings in the literature. Thus, no directional hypotheses were proposed. Further, as an exploratory aim, the possible role of immigrant status on the links between predictors and diversity trajectories was examined.

## Methods

### Participants

The sample for the current study was taken from a longitudinal study, the Three Cities Study, which aimed to identify the contributing factors and buffers common to various mental health problems among adolescents during the lower-secondary and high-school years. The Three Cities Study was conducted in 25 different schools (18 lower-secondary and 7 high schools) in neighborhoods with varying socio-demographic characteristics in three medium-sized cities in Sweden. The current study focused on the longitudinal sample that included students who were at grade 7 (age 13) during the first year of the study (T1). T1 data was collected in 2014. The students were re-assessed at grade 8 (T2) in 2015, and grade 9 (T3) in 2016.

Among the participating 7th grade students (*n* = 1457), only those with data on attitudes toward diversity at Time 1 were included, which gave an analytic sample of 1362 students (*M*_*age*_ = 13.18, *SD* = 0.43, 48% girls). A majority of the youth (68%) came from intact families, and had been living with both parents. About 19% of the youth had immigrant background. Immigrant background was defined as having both parents born outside of Sweden or another Nordic country (i.e., Finland, Norway, or Denmark). Among the immigrant youth, 40% were first-generation, and only 6% reported speaking Swedish at home with their parents. Two-thirds of them (75%) reported that they sometimes spoke Swedish and sometimes another language.

### Attrition Analysis

A logistic regression model was estimated to investigate whether attrition from T1 to T3 was due to any systematic bias. In the analytic sample (*n* = 1362), the dropout rate from T1 to T3 was 18% (*n* = 247). Attrition (dropout = 1, retention = 0) was regressed on the demographic characteristics of the adolescents (i.e., gender and immigrant background) and all the other study variables (i.e., openness to diversity, majority-minority friendship, friends’ views on diversity, classroom ethnic composition, and classroom climate). Adolescents’ immigrant background, their friends’ openness to diversity, and their perception of the classroom climate significantly predicted attrition (Nagelkerke *R*^2^ = 0.038). Specifically, adolescents of immigrant background, adolescents who had friends with low openness to diversity, and adolescents who perceived their classroom setting as less cooperative and socially cohesive were more likely to drop out of the study. Converting the Exp(B) values into Cohen’s *d* estimates to ease interpretation (Chinn 2000) revealed that the effect sizes of immigrant background (*d* = 0.31), friends’ openness to diversity (*d* = 0.03), and perceived classroom climate (*d* = 0.14) on attrition were low (Cohen 1988). Thus, it was concluded that attrition had only a minimal effect on the findings.

### Procedure

Research secretaries and trained test leaders attended the schools once a year from 2014 onwards, during the spring term, to invite adolescents to complete the questionnaire onsite during school hours. Trained test leaders administered the surveys, allowing students 90 minutes to complete the questionnaires and distributing snacks to participants during data collection. In addition, each class received 300 Swedish crowns in recognition of participation. Active consent from students and passive consent from parents were sought. Parents received a letter with information about the study, and a prepaid envelope with a form to be returned if they did not want their child to participate. Not returning this form was interpreted as giving consent (i.e., passive consent). This procedure for obtaining consent is frequently used in developmental studies to increase participation and reduce sampling bias (Pokorny et al. 2001; Shaw et al. 2015). The adolescents themselves gave their active consent by filling out the questionnaire after being informed that participation was voluntary. The study was approved by the regional ethics board of Uppsala (ref. number 2013/384). All data collection was carried out in accordance with the ethical principles of the Declaration of Helsinki.

### Measures

#### Openness to diversity

A four-item scale was developed to assess adolescents’ openness to diversity. The sample items include: “I accept and think I am open to other people who are very different than me,” “I feel comfortable talking with other people of my age who have opinions that are very different from mine,” “I am open to others who have different ways of doing things,” “I treat everyone equally, even if they are different from me.” Adolescents were asked to respond to each item on a 5-point scale, ranging from “1” (don’t agree at all) to “5” (agree totally). The measurement invariance of this scale over time was tested. The model where the items within each assessment point loaded on their own latent factors fitted the data well, *χ*^2^ (39) = 109.66, *p* < 0.001, CFI = 0.99, RMSEA = 0.04, *p* = 0.991, SRMR = 0.03, suggesting configural invariance of the measure over time. Then, the loadings were constrained to be equal to test the metric invariance. The model fitted the data well, *χ*^2^ (45) = 117.02, *p* < 0.001, CFI = 0.99, RMSEA = 0.03, *p* = 0.986, SRMR = 0.03, and the overall model fit did not differ from that of the freely estimated model, Δ*χ*^2^(6) = 7.36, *p* = 0.289. In sum, the findings indicated that the openness to diversity scale had metric invariance over time.

Scalar invariance of the measure at T1 across the groups of gender and immigrant background variables was also tested. The scalar invariance model with constraints on intercepts across the groups yielded an acceptable model fit. In addition, the differences between the scalar and metric invariance models were at or lower than the recommended cutoff value of −0.01 (Chen 2007) (CFI difference values: −0.01 for gender, and −0.007 for immigrant background), suggesting that the measure had scalar invariance across the groups of gender and immigrant background. Adolescents’ responses to the scale items were averaged to create the scale score. Inter-item reliability coefficients for this scale were good across the three assessment points (α = 0.85 at T1, α = 0.84 at T2, and α = 0.88 at T3).

#### Majority-minority friendship

Adolescents were asked to nominate three peers in their school whom they often spent time with, and did things together with (e.g., eating lunch or hanging out at breaks). A majority of the adolescents nominated classmates. In total, seventy-nine percent of them reported having three friends. About 11% of the adolescents did have missing data with regard to peer nomination. This percentage represents youth who did not nominate any friend *or* who nominated a friend whom it was not been able to identified. The data were restructured by placing both youth and their best friends’ answers to the survey questions in the same row. Then, the majority-minority friendship variable was created using adolescents’ immigrant background, and their friends’ immigrant background. The proportion of friends of immigrant background among Swedish adolescents, and the proportion of Swedish friends among immigrant youth, were estimated.

#### Friends’ openness to diversity

Following the procedure described in creating the “majority-minority friendship” variable, the data were restructured (i.e., the youth’s and their three friends’ answers on the openness to diversity scale were placed on the same row). Then, the scale scores on the openness to diversity measure of each of the three friends were computed. The friends’ openness to diversity measure was created by summing the scores of the three friends.

#### Classroom ethnic composition

Percentages of students with immigrant background were estimated across the participating classrooms (a total of 73), and these percentages were used to define classroom ethnic composition.

#### Perceived classroom social climate

A four-item scale was developed to measure how adolescents perceived the social climate in their classrooms. The items were: “We help each other in my class,” “We are nice to each other in my class,” “We like to do things together in my class,” and “No one feels left out.” Students responded to each item on a 5-point scale ranging from “1” (agree totally) to “5” (don’t agree at all). Adolescents’ responses to the scale items were reverse-coded and averaged to create the scale scores. The scale had very good inter-item reliability (α = 0.82 at T1).

### Data Analysis

A latent growth curve model in Mplus (Muthén and Muthén 1998–2010) was estimated to investigate how on average adolescents’ openness to diversity changed over time, and to examine whether there was significant variation in how adolescents perceived diversity. Then, latent class growth analysis was performed to identify whether there were distinct subgroups of adolescents who showed different patterns of change over time (Jung and Wickrama 2008). In order to identify the number of trajectory classes adequately, three recommended fit indices were used: the Bayesian information criterion (BIC, Kass and Raftery 1995), the classification accuracy (i.e., entropy, Muthén 2004), and the Lo-Mendell-Rubin adjusted likelihood ratio test (LMR-LRT, Lo et al. 2001). After identifying the final classes, multi-nominal logistic regression analysis was performed to examine whether adolescents’ gender, immigrant background, having a friend from the out-group, friends’ views on diversity, classroom ethnic composition, and adolescents’ perceptions of cooperation in the classroom influenced the developmental trajectory they followed over time. It was also explored whether the effects of the predictor variables (i.e., majority-minority friendship, friends’ views on diversity, classroom ethnic composition, and classroom social climate) varied across immigrant and Swedish youth. In order to estimate these effects, interaction terms were created between immigrant status and the predictor variables.

In the estimation of growth models (i.e., both latent growth curve and latent class growth models) and the multi-nominal logistic regression model, the clustering effect was controlled for by using the TYPE = COMPLEX command in Mplus to obtain corrected standard error estimates and unbiased test statistics, and to decrease the possibility of making a Type-I error. Of the analytical sample, 71.29% of the youth had full data on all study variables. Twenty-five missing data patterns were identified. Covariance coverage ranged between 0.74 and 1.00. These rates were substantially above the minimum criteria of 0.10 to reliably employ full information maximum likelihood (FIML) method to handle missing data. Thus, FIML method was used in the current study. It should be also noted that FIML has been shown to provide more reliable standard errors than mean imputation, or listwise or pairwise deletion (Little and Rubin 2002; Schafer and Graham 2002).

## Results

### Descriptive Statistics and Preliminary Analysis

Means, standard deviations, and correlations among the study variables are presented in Table 1. Female adolescents reported having more positive views on diversity than males, and they perceived their classroom environment as less cooperative and socially cohesive. Immigrant adolescents reported having less positive views on diversity than their Swedish peers. There was also a significant positive association between adolescents’ perception of the classroom social climate and their views on diversity, in that adolescents who perceived their classroom environment as more cooperative and socially cohesive were more likely to hold positive views on diversity. In addition, adolescents who had friends with greater openness toward diversity tended themselves also to have more positive views on diversity.

**Table 1:** Correlations, Means, and Standard Deviations for the Study Variables

	1	2	3	4	5	6	7	8	9
1. Gender	–								
2. Immigrant status	−0.03	–							
3. Majority-minority friendship at T1	−0.06^*^	0.43^***^	–						
4. Friends’ openness to diversity at T1	−0.12^***^	−0.16^***^	−0.10^***^	–					
5. Classroom ethnic composition at T1	−0.06^*^	0.54^***^	0.19^***^	−0.15^***^	–				
6. Classroom social climate at T1	0.09^**^	−0.04	−0.05	0.06	−0.08^**^	–			
7. Openness to diversity at T1	−0.15^***^	−0.18^***^	−0.05	0.11^***^	−0.12^***^	0.18^***^	–		
8. Openness to diversity at T2	−0.18^***^	−0.10^**^	0.03	0.10^**^	−0.08^**^	0.11^***^	0.48^***^	–	
9. Openness to diversity at T3	−0.19^***^	−0.05	0.01	0.08^*^	−0.09^**^	0.10^**^	0.29^***^	0.45^***^	–
M	–	–	0.13	9.16	0.21	3.78	3.62	3.86	4.01
SD	–	–	0.29	3.01	0.22	0.86	0.97	0.91	0.92

Gender was coded as: “1” boy and “0” girl. Immigrant status was coded as “1” immigrant and “0” Swedish

^*^p < 0.05; ^**^p < 0.01; ^***^p < 0.001

### **Do**es Adolescents’ Openness to Diversity Change over Time?

A latent growth curve model was estimated to examine how adolescents’ views on diversity changed during adolescence (from 13 – 15 years). A growth model was fitted, where the shape of change was defined as linear, which was achieved by fixing the time scores for the slope growth factor to 0, 1, and 2. The model fitted the data well, χ^2^(1) = 2.15, *p* = 0.14, CFI = .99, RMSEA = 0.029, SRMR = 0.013. The mean of the slope was positive and statistically significant (*M*_*slope*_ = 0.18, *z* = 10.49, *p* < 0.001), indicating that the adolescents, on average, became more open toward diversity as they got older. Furthermore, the variances of both intercept and slope were statistically significant (*s*^*2*^_*intercept*_ = 0.59, *z* = 10.86, *p* < 0.001; *s*^*2*^_*slope*_ = 0.14, *z* = 5.57, *p* < 0.001), suggesting that there were significant inter-individual differences in the level of adolescents’ openness to diversity at T1 and in how adolescents’ attitudes changed over time. Together, these findings suggest that there might be unique subgroups of adolescents who follow different growth trajectories.

### Trajectories in Adolescents’ Openness to Diversity

In order to examine whether there were unique subgroups of adolescents who follow different growth trajectories regarding their views on diversity, latent class growth models with different cluster solutions were estimated. The fit statistics for all class solutions are presented in Table 2. As shown in Table 2, the two-, three-, and four-class solutions showed improvements in AIC and BIC. The results of the LMR-LRT were statistically significant for the two-class and three-class solutions. However, the LMR-LRT was not statistically significant for the four-class solution, which indicated that moving from a three-class to a four-class solution did not improve model fit. Thus, the three-class solution was retained as the one that fitted the data best. In addition, the model parameters for the three-class solution were replicated using the OPTSEED command. This suggested that the final cluster solutions were robust.

**Table 2:** Latent Class Growth Model Fit Indices for Views on Diversity

Class	AIC	BIC	Entropy	LMR-LRT
1 Class	9400.78	9442.48	–	–
2 Class	9453.18	9494.87	0.70	0.001
3 Class	9315.92	9373.25	0.68	0.02
4 Class	9210.99	9283.96	0.73	0.45

Sixty percent of the sample (*on a high-increasing trajectory*) had high levels of openness to diversity at grade 7 and became even more open from grade 7 to grade 9. Thirty-four percent of the adolescents (*on an average-increasing trajectory*) reported average levels of openness to diversity at grade 7, and became more open over time. Finally, about 6% of the adolescents (*on an average-declining trajectory*) reported an average level of openness to diversity at grade 7, but sharply decreased in their openness over time.

**Fig. 1:**
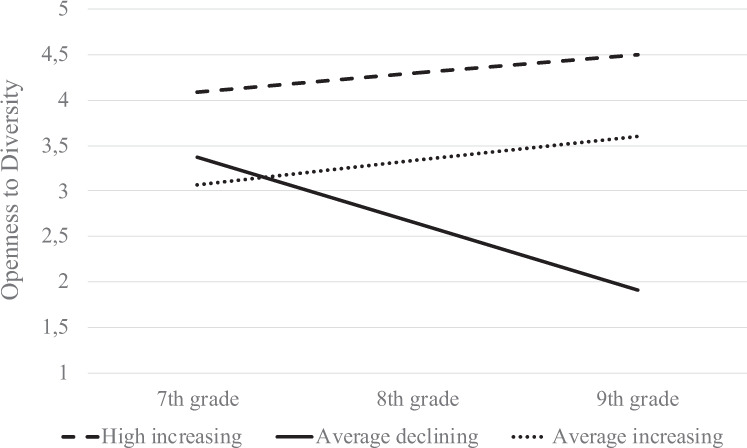
Openness to diversity trajectories

The three latent clusters of adolescents were compared on their level of openness to diversity across the three time points. The results suggested that adolescents on a high-increasing trajectory had higher levels of openness to diversity than those on average-increasing and average-declining trajectories, but that there was no significant difference between those on an average-increasing and an average-declining trajectory at T1, *F*(2, 1353) = 308.80, *p* < 0.001. The three clusters differed significantly in their openness to diversity at T2, *F*(2, 1229) = 535.65, *p* < 0.001, and at T3, *F*(2, 1095) = 1137.60, *p* < 0.001. The estimated mean trends for the three trajectories are presented in Fig. 1.

### Predictors of Openness to Diversity Trajectories

A multinomial logistic regression analysis was conducted to see whether adolescents’ demographic characteristics (i.e., gender and immigrant status), majority-minority friendship, friends’ views on diversity, classroom ethnic composition, and youth’s perceptions of classroom climate predicted classification into any one of the latent trajectory classes. In this model, adolescents in the high-increasing group were defined as the reference group, given that they were the normative developmental group. This approach allows examination of what predicts being on either the average-increasing or the average-declining trajectory compared with the high-increasing trajectory.

The results showed that adolescents in the average-increasing group had a higher likelihood of being male and having an immigrant background than those in the high-increasing group. In addition, these adolescents perceived their classroom climate as less cooperative and socially cohesive than those in the high-increasing group. Having an out-group friend did not significantly differentiate the youth in the average-increasing group from those in the high-increasing group. There were also no significant main effects of friends’ views on diversity and classroom ethnic composition (see Table 3).

**Table 3:** Multinomial Logistic Regression Results

	B	*z*	OR	*p*
*Average-increasing*				
Gender	0.79	6.46	2.20	0.001
Immigrant status	0.71	3.71	2.04	0.001
Majority-minority friendship	−0.36	−1.46	0.70	0.146
Friends’ views on diversity	0.02	0.67	1.01	0.501
Classroom ethnic composition	0.25	0.72	1.29	0.469
Classroom social climate	−0.49	−6.29	0.61	0.001
*Average*-*declining*				
Gender	1.64	5.08	5.14	0.001
Immigrant status	0.80	2.43	2.22	0.015
Majority-minority friendship	−0.50	−1.09	0.61	0.278
Friends’ views on diversity	−0.11	−2.73	0.89	0.006
Classroom ethnic composition	1.02	2.16	2.76	0.031
Classroom social climate	−0.19	−1.28	0.83	0.201

Adolescents in the high-increasing group were defined as the reference group. Gender was coded as: “1” boy and “0” girl. Immigrant status was coded as “1” immigrant and “0” Swedish

The results also revealed that adolescents in the average-declining group had a greater likelihood of being male and having an immigrant background than adolescents in the high-increasing group. Importantly, friends’ views on diversity significantly differentiated the youth in the average-declining group from those in the high-increasing group. Specifically, adolescents who had friends with positive views on diversity were less likely to be in the average-declining group than those in the high-increasing group. The main effect of classroom ethnic composition was also statistically significant, showing that adolescents in ethnically heterogenous classrooms were more likely to be in the average-declining group than in the high-increasing group. Neither having an out-group friend nor youth’s perception of classroom social climate significantly differentiated the youth in the average-declining group from those in the high-increasing group (see Table 3).

As a secondary analysis, it was also tested whether adolescents’ peer (i.e., majority-minority friendship, friends’ openness to diversity) and classroom contexts (i.e., classroom ethnic composition and perception of classroom social climate) predicted membership in latent trajectory classes depending on their immigrant status. Several interaction terms between immigrant status and these predictor variables were created and entered as predictors in the multinomial logistic regression model. The results revealed only one significant interaction effect. Specifically, it was found that immigrant youth who have friends with positive views on diversity were less likely to be in the average-increasing group than in the high-increasing group, *B* = −0.27, *z* = −3.12, OR = 0.76, *p* = 0.002. This effect was not observed among Swedish youth, *B* = −0.09, *z* = −1.68, OR = 0.92, *p* = 0.09. Altogether, these findings suggest that adolescents follow different pathways in their views on diversity as they get older. Adolescents’ gender, immigrant background, socialization context and experiences in school all seem to play a role in determining the trajectories they follow.

## Discussion

Adolescents undergo major developments in how they think about themselves and members of out-groups. These developments may have enduring effects on how they develop into adults, view diversity, and interact with people from other ethnic and cultural groups. Increasing ethnic and cultural diversity in schools stimulates research that elucidates the development of youth’s attitudes toward diversity and the factors that contribute to this development. Accordingly, the present study examined how adolescents’ views on diversity change during the secondary school years, and the extent to which their demographic characteristics and the social context in school contribute to the process of change.

The findings suggest that adolescents, on average, display a linear increase in their positive views on diversity during the secondary school years. This finding is consistent with previous studies (van Zalk and Kerr 2014; Wölfer et al. 2016), which show a linear increase in tolerant attitudes toward immigrants from early to late adolescence. It is also in line with the developmental change that occurs during adolescence. Specifically, the adolescence period is marked by developing cognitive maturity and improving perspective-taking skills (despite heterogeneity across individuals) (Eisenberg et al. 2005). These developmental changes may help youth look beyond their own points of view, and, in turn, help them find reasons why, for example, their peers may think, behave, or live in different ways than they do. Such understanding may lead them to tolerate differences rather than perceiving them as potential threats.

A noteworthy finding of the present study is that not all adolescents change in their views on diversity in the same direction. Three distinct subgroups were identified. Specifically, more than half of the adolescents in the study (60%) had high positive views on diversity when they were 13 years-old, and continued to increase in their positive views over time. About one-third of them (34%) started off with an average level of positivity and became more open to diversity as they got older. A small but significant percentage of the adolescents (6%) had fairly positive attitudes toward diversity at the beginning of secondary school, but their positive attitudes declined over time. Together, these findings suggest that a majority of young people follow the expected developmental trend in their views on diversity. That is, they hold positive views toward diversity at age 13 and have become even more positive by the time they reach age 15. However, a small group of adolescents show a decline in their positivity, which is a cause for concern.

Another important aspect of this study is its examination of possible factors that may illuminate why young people follow different trajectories. The present research draws attention to the importance of peer context in the formation of adolescents’ attitudes. Supporting previous research (Bayram Özdemir et al. 2018; Miklikowska 2017; van Zalk et al. 2013), it was found that adolescents tend to share views with their peers. Specifically, it was shown that adolescents whose friends have positive views on diversity are more likely to be on the high-increasing trajectory than those on the average-increasing and the average-declining trajectories. One possible explanation for this is that adolescents who are surrounded by open-minded friends have the opportunity to socialize in a non-judgmental setting and be exposed to differing views. Such a context may help them move away from a self-centeredness in their views, and see differences as a way to gain new perspectives.

Interestingly, majority-minority friendship did not predict how adolescents changed in their views on diversity. This finding is out of line with the contact hypothesis (Allport 1954) and previous empirical findings (Pascarella et al. 1996; Whitt et al. 2001), both of which indicate that the more young people interact with diverse peers, the more they become open to diversity. Three possible explanations can be proposed for the lack of effect in the current study. First, as indicated previously, inter-ethnic contact may be contextually bounded in its effect on young people’s views and attitudes. That is, having an out-group friendship might help young people to develop positive views on a specific outgroup, but it might not have the spillover effect of promoting adolescents’ openness to diversity in general. Second, unlike in previous research that has not controlled for friends’ attitudes (Pascarella et al. 1996; Whitt et al. 2001), in the present study, the effect of majority-minority friendship was examined after considering peers’ views on diversity. Our findings clearly show that friends’ views may be more of an influence than the countries from which they come. Being surrounded by open minded friends, regardless of their backgrounds, may enhance openness to diverse perspectives in adolescents. A third explanation may be related to the kinds of people with whom the adolescents have cross-ethnic friendships. Friendship with someone from a culturally distant group may have a different influence on an adolescent’s experiences and openness to diversity than friendship with one who is from a culturally close group. The study’s data enabled identification of whether a youth in the sample had an immigrant background or not, but no information was available on, for example, which country the immigrant youth came from. This lack of data limited capacity to examine majority-minority friendship in more specific terms (e.g., ethnic, religious, or with regard to cultural background), and, in turn, to draw more robust conclusions.

Another important conclusion to draw from the findings is that adolescents’ perceptions of the classroom climate seem to play a role in how their views on diversity change over time. Specifically, it was found that adolescents who perceive their classroom climate as cooperative and socially cohesive are more likely to be on the high-increasing trajectory than those on the average-increasing trajectory. This finding is in line with previous research (Gniewosz and Noack 2008; Isac et al. 2012), suggesting that creating a classroom environment where students have the opportunity to collaborate and interact with each other harmoniously may be a key to promoting the tolerance of differences. As previously stated, a socially cohesive classroom environment may facilitate the development of “we-ness” and, in turn, students in such an environment may not perceive difference as a threat toward themselves, but rather an opportunity to learn more about new perspectives. School personnel, particularly teachers, have a critical role to play in the formation and maintenance of socially inclusive and collaborative classroom environments. However, not all teachers feel competent to handle issues related to diversity in school (Bayram Özdemir et al. in press). Thus, teachers’ skills in working effectively in diverse classrooms might need to be promoted by providing in-service training. Such systematic support might in the end contribute to the promotion of positive interactions between diverse groups of students.

The results show that males have a greater likelihood than females of being on the average-declining trajectory. This finding is in line with previous studies showing that males on average are less likely to be open to diversity (Pascarella et al. 1996), and have lower tolerance of disadvantaged groups (Tucker Smith et al. 2016) and immigrants (Akrami et al. 2000) than females. It also extends the previous literature by showing that male adolescents are at greater risk of becoming intolerant of differences over time. This may be related to differences in emotional and cognitive skills found between males and females; for example, the literature consistently shows that females are better at perspective-taking (Tucker Smith et al. 2016) and have greater empathic concerns (Butrus and Witenberg 2013). Thus, it is possible that the observed gender differences in openness to diversity are due to individual differences in perspective-taking skills or empathic concerns between males and females.

The findings also indicate that adolescents’ immigrant background plays a role in how they change in their views on diversity over time. Specifically, adolescents of immigrant background were found to have a greater likelihood of being on the average-declining trajectory than those of non-immigrant background. This finding runs contrary to previous research (van Zalk et al. 2013), which has shown that immigrant adolescents increase more in their tolerance than those of non-immigrant background in Sweden. One explanation for the contradictory findings could be related to variation in the operationalization of constructs across the studies. In the current study, openness to diversity was operationalized as “an awareness and potential acceptance of both similarities and differences in others” without referencing any specific group. However, the previous research focused on adolescents’ tolerant attitudes specifically toward immigrants (van Zalk et al. 2013). It is possible that immigrant adolescents might have positive biases toward “immigrants” because they associate themselves with this societal category. Being in the same category, they may develop more favorable attitudes toward other immigrants. Together, the findings suggest that promoting tolerance of differences between native and immigrant youth may not be achievable by focusing on just one side of the coin, i.e., on majority youth. There is a need to develop understanding of why immigrant youth are less likely to be open to diversity, and what factors explain why they may be at risk of becoming more intolerant over time.

Despite its important contributions to the literature, the present study has several limitations and has left some important issues unattended. First, the current study used 3-year longitudinal data. Although an important methodological strength, this meant that, like most longitudinal studies, it suffered from attrition. Despite the low rate of attrition, analysis showed that drop-out rates were higher among adolescents of immigrant background, among adolescents with non-open-minded friends, and among adolescents who perceived their classroom setting as less cooperative. Thus, the findings of the current study need be interpreted cautiously by taking this into account. Second, the present study was guided by reasoning rather than empirical analysis in selecting predictor and outcome variables, and forming study hypotheses. Its primary focus was on examining socialization and/or contextual effects on youth’s views on diversity. It should, however, be acknowledged that a selection effect may well be present. That is, youth who are open to diversity may be more likely to have friendships with outgroup members and socialize with open minded friends. Future studies are needed to test alternative explanations. Third, the study focused on the general social climate of the classroom. However, other aspects of school context, including varieties of multicultural education and teachers’ approaches to diversity, may also play a role in how adolescents diversity beliefs develop and change over time. In fact, in a study focusing on university students, it was shown that young adults taught by faculty members who advocate that their students respect diverse viewpoints are more likely to be open to diverse perspectives (Ryder et al. 2016). Thus, future research tapping into different aspects of the school context is needed to better understand the role of school context in youth’s views on diversity. Fourth, the focus of this study was primarily on the school setting (i.e., peers and classroom social climate). However, not only the school itself but also parents, may play an important role in how adolescents view others who are different from themselves. Examining the unique effects of both contexts, and also their interactive effects, may advance understanding of openness to diversity. Finally, the present study was concerned with understanding the possible underlying reasons why youth follow different trajectories. However, it does not provide any information on how the youth who were classified into the different trajectories of openness to diversity behave in diverse settings. Examining the inter-ethnic behaviors of youth who follow different developmental trajectories (e.g., racist behaviors, radicalization, ethnic victimization) would further understanding of social inclusion and racism.

## Conclusion

During adolescence, young people seek to develop and deepen their understanding of the world, and form opinions and attitudes. They also become increasingly independent of their parents, and are susceptible to new influences and experiences. All these changes make adolescence a window of opportunity for understanding the formation of views on diversity and behaviors in diverse settings. Considering these developmental changes, the present study attempts to shed light on how young people’s views on diversity emerge and change during adolescence. The findings clearly suggest that adolescents, on average, become more open to diverse perspectives over time. There is, however, clear heterogeneity among adolescents on how their views change over time. The findings show that males and youth of immigrant background are at risk of becoming less open to diverse perspectives. To date, most research on openness to diversity has been concerned with the views of the majority group regarding minorities or immigrants. The current findings indicate the importance of adopting a holistic perspective, by not only focusing on majority youth (which is the common approach), but also developing an understanding of why minority youth are more at risk. The findings also suggest that schools may serve as a common ground for promoting openness to diversity. Establishing a classroom context where adolescents help each other and cooperate in day-to-day activities may provide them with an opportunity to interact and work on common goals with peers who are different from themselves. Such an opportunity may help adolescents see the similarities between themselves and their peers, while at the same time perceiving differences as paths to new perspectives rather than as possible threats.
